# Process Parameters Optimization, Characterization, and Application of KOH-Activated Norway Spruce Bark Graphitic Biochars for Efficient Azo Dye Adsorption

**DOI:** 10.3390/molecules27020456

**Published:** 2022-01-11

**Authors:** Marine Guy, Manon Mathieu, Ioannis P. Anastopoulos, María G. Martínez, Frédéric Rousseau, Guilherme L. Dotto, Helinando P. de Oliveira, Eder C. Lima, Mikael Thyrel, Sylvia H. Larsson, Glaydson S. dos Reis

**Affiliations:** 1 National Chemical Engineering Institute in Paris, 75005 Paris, France; marine.guy@etu.chimieparistech.psl.eu (M.G.); frederic.rousseau@chimieparistech.psl.eu (F.R.); 2 Université de Toulouse, Mines Albi, CNRS UMR 5302, Centre RAPSODEE, 81000 Albi, France; manon.mathieu@mines-albi.fr (M.M.); maria.gonzalez_martinez@mines-albi.fr (M.G.M.); 3 Department of Agriculture, University of Ioannina, UoI Kostakii Campus, 47040 Arta, Greece; anastopoulos_ioannis@windowslive.com; 4 Chemical Engineering Department, Federal University of Santa Maria (UFSM), Santa Maria 97105-900, RS, Brazil; guilherme_dotto@yahoo.com.br; 5 Institute of Materials Science, Federal University of Sao Francisco Valley, Juazeiro 48920-310, BA, Brazil; helinando.oliveira@univasf.edu.br; 6 Institute of Chemistry, Federal University of Rio Grande do Sul, Porto Alegre 91501-970, RS, Brazil; profederlima@gmail.com; 7 Department of Forest Biomaterials and Technology, Swedish University of Agricultural Sciences, 90183 Umeå, Sweden; mikael.thyrel@slu.se (M.T.); sylvia.larsson@slu.se (S.H.L.)

**Keywords:** biomass, porous material, Evans blue dye, design of experiments, kinetic and equilibrium study

## Abstract

In this work, Norway spruce bark was used as a precursor to prepare activated biochars (BCs) via chemical activation with potassium hydroxide (KOH) as a chemical activator. A Box–Behnken design (BBD) was conducted to evaluate and identify the optimal conditions to reach high specific surface area and high mass yield of BC samples. The studied BC preparation parameters and their levels were as follows: pyrolysis temperature (700, 800, and 900 °C), holding time (1, 2, and 3 h), and ratio of the biomass: chemical activator of 1: 1, 1.5, and 2. The planned BBD yielded BC with extremely high SSA values, up to 2209 m^2^·g^−1^. In addition, the BCs were physiochemically characterized, and the results indicated that the BCs exhibited disordered carbon structures and presented a high quantity of O-bearing functional groups on their surfaces, which might improve their adsorption performance towards organic pollutant removal. The BC with the highest SSA value was then employed as an adsorbent to remove Evans blue dye (EB) and colorful effluents. The kinetic study followed a general-order (GO) model, as the most suitable model to describe the experimental data, while the Redlich–Peterson model fitted the equilibrium data better. The EB adsorption capacity was 396.1 mg·g^−1^. The employment of the BC in the treatment of synthetic effluents, with several dyes and other organic and inorganic compounds, returned a high percentage of removal degree up to 87.7%. Desorption and cyclability tests showed that the biochar can be efficiently regenerated, maintaining an adsorption capacity of 75% after 4 adsorption–desorption cycles. The results of this work pointed out that Norway spruce bark indeed is a promising precursor for producing biochars with very promising properties.

## 1. Introduction

Carbon-based materials are commonly used in wastewater treatment, soil amendment, gas emission mitigation in the greenhouse, chemical catalysts, and energy storage systems [1,2,3]. Their several remarkable properties, such as highly developed porosity and internal pore structure, elevated specific surface area, and a high degree of surface chemistry, make carbon-based materials efficient candidates for these applications [3,4].

Lately, several carbon-based materials, such as activated carbons, biochars, graphene, carbon nanotubes, and composites of these materials, have been utilized as adsorbents [1,4,5]. Activated carbon is the most utilized adsorbent; however, the use of biomass for producing biochar (BC) has led to biochars gaining attention as adsorbents in recent years [1,2].

Biochars are efficient for use in wastewater treatment, primarily due to their highly porous structure and their numerous chemical functionalities [1,2,3,4]. In wastewater treatment processes, adsorption is preferred due to characteristics of low implementation costs, relative operation simplicity, and minimal generation of by-products [4,5,6,7]. 

Pyrolysis is the most common process for producing BCs. However, the efficiency of the pyrolysis process in BC yielding with improved properties is influenced by several factors, such as pyrolysis temperature, holding time, and heating rate [4]. Therefore, the efficient control of these parameters is required to produce BCs with improved textural properties [1,2,4]. Additionally, the preparation of BC has another critical step, the activation process, which can be either physical or chemical [1,2,5,6]. Chemical activation is performed in the presence of chemical agents. It has the advantage of generally being conducted in a one-step process, using lower activation temperatures, generating BC with higher SSA values, and is a more straightforward process to adjust the porosity of the BCs [6,7]. However, some disadvantages of using chemical activation are reported, as the chemical reagents are non-environmentally friendly substances and processes, such as in the acidic washing step, which is applied to remove the remaining chemical activating agent in the carbonized matrix. 

Several chemical agents are used to impregnate the raw materials, among which, zinc chloride (ZnCl_2_) and KOH have been considered the most common ones. The BC produced with ZnCl_2_ yields materials with developed mesoporosity, while KOH activation generally creates microporous and small mesoporous structures with higher SSA [8,9]. Therefore, the characteristics of the BC can also be adjusted according to the chemical activation employed. Thus, the KOH activation and its effects on BC characteristics were explored in this work. 

For the production of BC, the isolated and combined influence of variables can play an essential role in the process and may introduce additional difficulties to identify and understand at what condition the BCs with improved properties are produced. For this purpose, statistical designs of experiments have been considered helpful tools that are successfully employed to evaluate the influence of the combined variables in the process. The response surface methodology (RSM) is a valuable statistical tool to optimize different variables utilized to obtain better characteristics of the desired material [4,10]. The application of RSM is critical for developing a cost-effective process by elucidating which experimental variables affect BCs’ properties, and how their conditions function [9,10]. The RSM aims to reduce the costs and maximize the operation process efficiency over a minimal number of experimental runs [11]. RSM is a proven method that has been widely applied in the literature for the optimization of experimental conditions for BC preparation [10,11,12,13,14,15].

Azo dyes are organic compounds applied in several domains, such as the food, biomedical, and textile industries. They are commonly found in the wastewater of these industries, with their high solubility in water, and are dangerous to the environment and human life [16,17]. Evans blue (EB) dye, also named Direct Blue 53 dye, belongs to the azo dye class, and its degradation may lead to mutagenicity and carcinogenicity in by-products, especially for the human fetus [18,19]. Furthermore, EB has been considered a toxic dye at high dosages, provoking chronic health effects—affecting lung, liver, and intestine function [20]. Therefore, treating effluents loaded with dyes is a vital activity before discharging them into the environment.

The main goal of this study is to determine the effect of pyrolysis conditions on the properties of the spruce bark BCs. In order to optimize these results, an RSM assay was employed, in which variables (pyrolysis temperature, holding time, and the ratio of biomass: KOH) were studied over two responses: SSA and mass yield. Based on the RSM results, the BC with the highest SSA was chosen and employed to remove Evans blue dye by adsorption process, from which the effect of initial pH, kinetic and equilibrium studies were evaluated. The adsorption–desorption (cyclability) cycles were also studied. Finally, the efficiency of the BC to remove several dyes in synthetic wastewater was evaluated. 

## 2. Results and Discussion

### 2.1. Process Parameters Optimization for SSA and Yield

The SSA is an important property of carbon materials due to its massive influence on the desired application, i.e., adsorption performance in removing pollutants from polluted waters [1,5,14]. The SSA and mass yield results of each sample are shown in Table 1. Table 1 shows the results of 15 experimental runs, obtained through Box–Behnken design, by evaluating 3 variables (pyrolysis temperature, holding time, and ratio of activating agent) on 2 responses (SSA and Yield).

The data presented in Table 1 show that the SSA values ranged from 274 to 2209 m^2^. g^−1^, confirming that Norway spruce bark can be considered an efficient precursor for producing BC with very high SSA values. For instance, Leite et al. [9] produced BCs from avocado seed and reported SSA values in the range of 1122–1584 m^2^·g^−1^. Dos Reis et al. [10] prepared BCs using sludge sewage as the precursor and reported SSA values up to 679 m^2^·g^−1^.

To further compare our results with the literature, Table 2 presents a short review, containing other studies regarding bio-based activated carbons made with KOH [21,22,23,24,25]. Yagmur et al. [21] used Oleaster fruits’ flesh to make BC with the highest SSA equal to 1816 m^2^·g^−1^ at pyrolysis temperature of 800 °C and holding time of 1 h, with a biomass: KOH ratio of 1:3. Guclu et al. [22] employed spent tea leaves to make BC with SSA in the order of 820.7 m^2^·g^−1^ at pyrolysis temperature of 800 °C and holding time of 1 h, with a biomass: KOH ratio of 1:1. 

According to these results, it can be stated that Norway spruce bark, under the right preparation conditions, is a very suitable precursor for producing highly porous materials. In addition, it is possible to infer that the optimum pyrolysis condition is dependent of the type of the biomass precursor characteristics, and how the pyrolysis process was performed. Therefore, more profound studies are necessary to reliably obtain at which pyrolysis conditions the bio-based carbon materials with improved characteristics are produced. The use of RSM to evaluate and identify which pyrolysis parameters influenced the SSA values is a valuable alternative. Therefore, the following section will evaluate the influence of the pyrolysis conditions on the SSA values of the BCs, using RSM.

#### Parameters Optimization for SSA and Yield of the BC

RSM analysis can be explored to obtain the most influential parameters, for the BC preparation, over the selected responses, SSA, and yield of BCs. Through RSM, it is possible to find the optimized parameters to produce BC with the high SSA values and mass yield.

**Figure 1:**
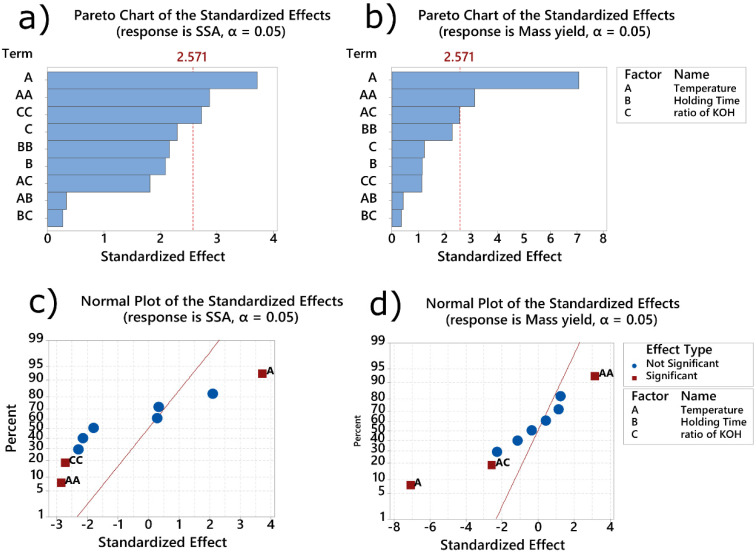
Normal plot for standardized effects: (**a**) SSA; (**b**) mass yielding. Pareto chart for: (**c**) SSA; (**d**) mass yielding.

The effect of the A (pyrolysis temperature, °C), B (holding time (min)), and C (ratio of biomass: KOH) on SSA values is shown in Figure 1 (Pareto chart and normal plot). The Pareto chart (see Figure 1a,b) indicates that pyrolysis temperature (A) and the quadratic term of temperature (A^2^) and the quadratic term of ratio (C^2^) were the factors of influence for the SSA. When the response was yielding mass values, the parameters A (temperature), A^2^ (quadratic of temperature), and A.C (the combination of temperature versus the ratio of activating agent) were the factors that influenced the mass yielding. The normal plot in Figure 1c,d) indicates that temperature influenced the SSA value positively, whereas the terms A^2^ and C^2^ had a negative effect on the SSA.

For the response mass yielding, the term A^2^ had a positive effect on the response; on the other hand, the term A and A.C had a negative effect on the mass yielding. Thus, it is remarkable to show that the statistical response surface methodology helps one arrive at the best condition of optimization of the parameters, and one term of the RSM should not be independently evaluated. For example, for the response SSA, the increase in temperature leads to an increase in the response; however, the term A² (quadratic of temperature) leads to a decrease in SSA. The same trend was observed for the yielding mass response. The increase in temperature led to a decrease in the mass yielding; however, the term A² leads to an increase. This observation is possible using statistical response surface methodology and could not be achieved using one-factor optimization (univariate optimization).

**Figure 2:**
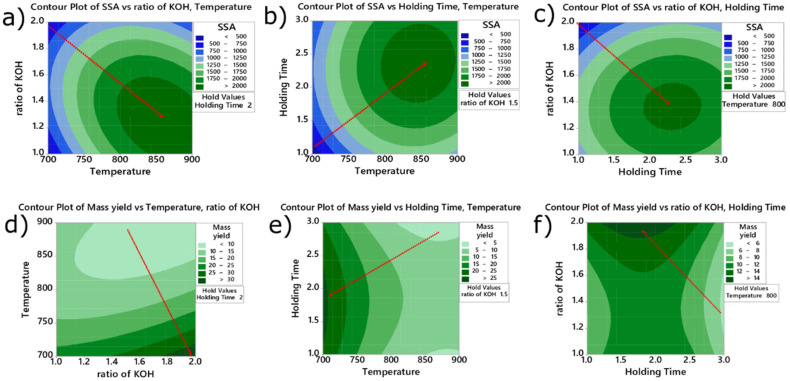
Contour plots of SSA and mass yielding versus each pair of parameters. Contour Plot of SSA vs. ratio of KOH, Temperature (**a**); Contour Plot of SSA vs. Holding Time, Temperature (**b**); Contour Plot of SSA vs. ratio of KOH, Holding (**c**); Contour Plot of Mass yield vs. Temperature, ratio of KOH (**d**); Contour Plot of Mass yield vs. Holding Time, Temperature (**e**); Contour Plot of Mass yield vs. ratio of KOH, Holding Time (**f**).

The effect of the variables of the BC preparation on SSA and mass yielding values is further studied through contour plots (shown in Figure 2). This plot makes it possible to observe the behavior of the variables’ levels where a region with higher values can be found (represented by dark green areas). In addition, dashed arrows in red were inserted to show the maximum response on SSA and mass yielding to facilitate the visualization. 

According to Figure 2a–c (SSA as a response), the highest SSA values can be obtained at high temperatures but with a low ratio of KOH. Regarding SSA for holding time versus temperature, the holding time should be between 2 h 30 min and 2 h 45 min, the response for ratio versus time, and the ratio between 1.25–1.50. When the response was the ratio of biomass: activating agent, (Figure 2d–f), the maximum mass yielding took place when the temperature was lower (700 °C—see Figure 2d,f) using a ratio of biomass: activating agent of 1:2 (see Figure 2e) and holding time of 1.5–2.0 h (Figure 2e).

**Figure 3:**
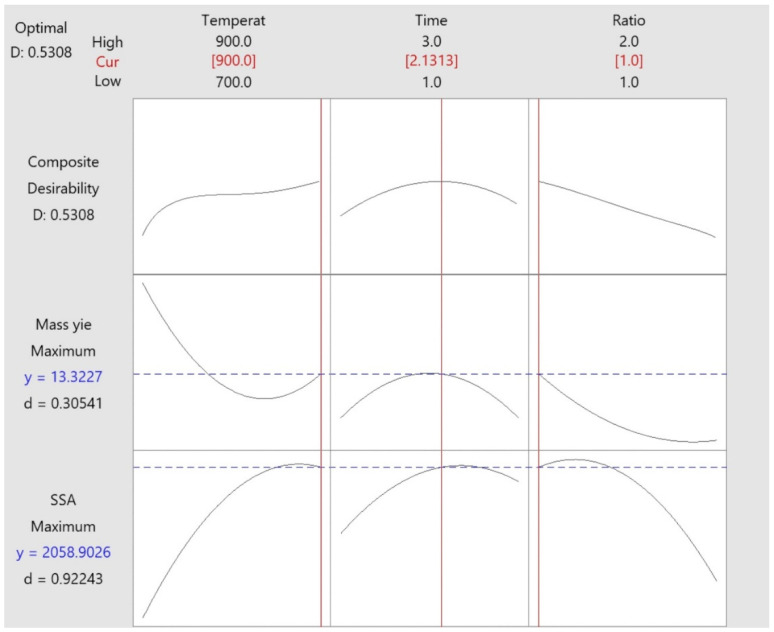
Optimization of the response mass yielding and SSA for production of BC.

The surface area is a factor that is crucial for obtaining adsorbents with high sorption capacity. Adsorbents that present high surface areas usually present high sorption capacities to remove different species [3,4]. From the economic point of view, the yield is an important parameter that can be a decisive issue in the industrial scale-up process. Therefore, an optimization should involve the two responses, surface area, and mass yielding. Figure 3 presents a graph that is the optimization of both responses.

The overall desirability for optimization of the maximum SSA and mass yielding of produced BC responses was 0.5308. According to this optimization, it would be recommended to use a holding temperature of 900 °C, a holding time of 2.13 h, and a ratio of biomass: KOH of 1:1.

### 2.2. BCs Characterization

#### 2.2.1. Textural Properties and Morphology of BCs

Specific surface area and porosity are essential properties that play a strong influence on the performances of the BC regarding its application, e.g., as an adsorbent for removing pollutants from residual water [1,5,14]. Therefore, four samples (BC6, BC4, BC11, and BC7) were chosen to evaluate these properties. The choice was based on the highest and lowest SSA values and intermediate values.

**Figure 4:**
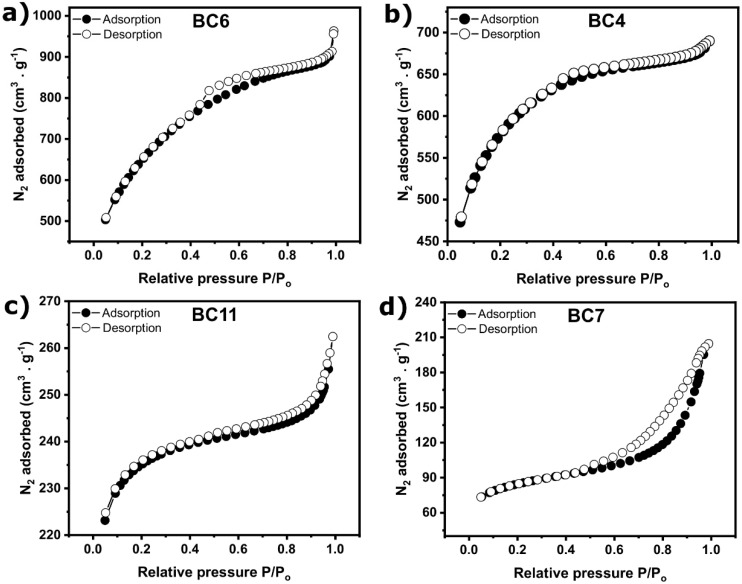
Nitrogen adsorption–desorption isotherms of (**a**) BC6, (**b**) BC4, (**c**) BC11, and (**d**) BC7.

Nitrogen adsorption–desorption isotherms of the four samples are presented in Figure 4. According to the IUPAC classification [26], isotherms of BC6 and BC4 are close to type I as the nitrogen adsorption increases at low partial pressure. This type describes an adsorption process resulting in micropores filling. As a consequence, BC6 and BC4 should contain certain quantities of micropores. However, both curves did not reach a limiting value, and the shape of isotherms at high partial pressure is closer to type IV isotherms. This type is typical of a mesoporous material. Moreover, a small H4 hysteresis loop is seen on BC6’s isotherm, which indicates that the structure is mesoporous and microporous [26].

On the other hand, BC11 and BC7 show a type IV isotherm (characteristic of mesoporous materials). In addition, the hysteresis is very distinct on BC7’s curve, which indicates that mesopores are wider than 4 nm, while it is not distinct at all for BC11′s isotherm because mesopores are small. Moreover, according to Figure 4c,d, BC11 and BC7 also adsorb N_2_ at low partial pressure, which means they should present a combination of micropores and micropores in their structure. 

**Figure 5:**
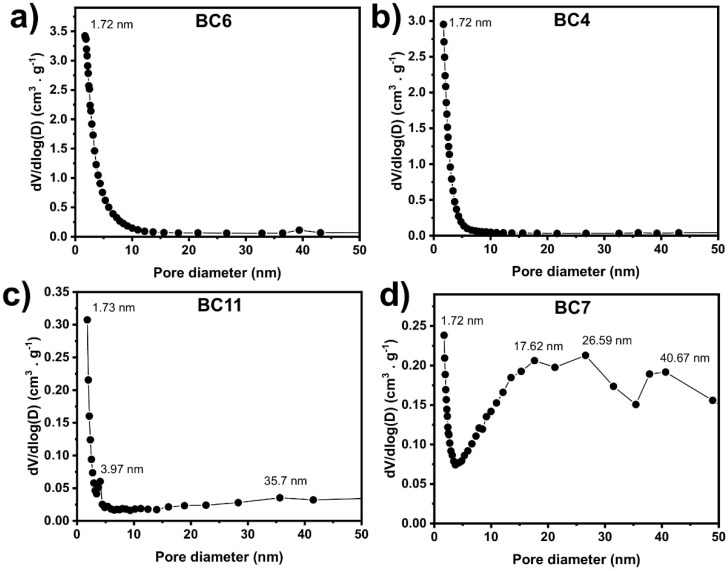
Pore size distribution of (**a**) BC6, (**b**) BC4, (**c**) BC11, and (**d**) BC7.

The porosity of these four samples has been further studied by plotting the pore size distribution curves (see Figure 5). The four samples contain a rich structure of micropores with a pore diameter of 1.72 nm. The highest micropores are observed for sample BC6, despite small mesopores with a diameter between 2 and 10 nm. The volume of micropores is higher for BC6 and BC4, which have a higher specific surface area than BC11 and BC7. BC7’s pore size distribution curve indicates the presence of wide mesopores with a diameter between 17.6 nm and 40.7 nm. These results are in agreement with the results observed in the nitrogen adsorption isotherms.

Results on microporosity and mesoporosity contributions in the BC are summarized in Table 3. As can be seen in Table 3, a high amount of microporous is observed in all samples. As indicated by S_micro_ and S_micro%_ values, the spruce bark BCs showed to be composed of more micropores; for instance, the percentages of micropores (with relation to SSA values) of BC6, BC4, BC11, and BC7 were 77.4%, 56.3%, 86.4%, and 54.7%, respectively. However, large quantities of mesopores are also observed, especially for the samples BC7 (43.7%) and BC6 (45.3%). Micropores and mesopores are highly desirable in BCs for adsorption because they ensure wetting and liquid transport throughout the bulk of the BC.

**Figure 6:**
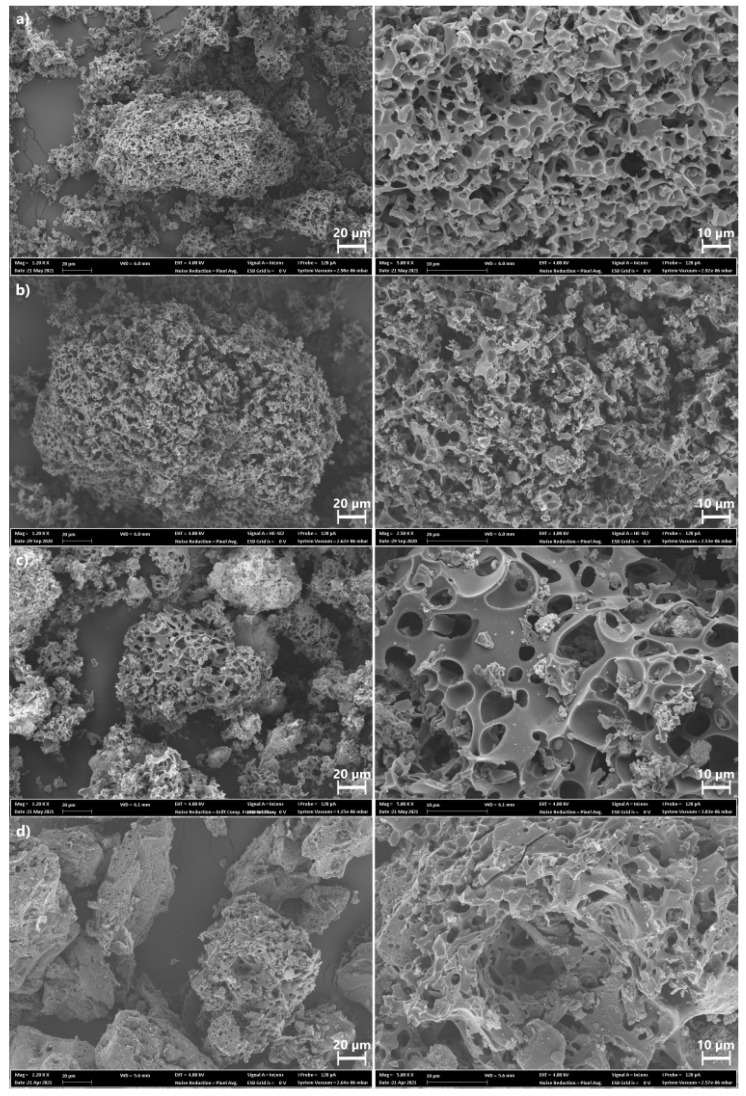
SEM images at a 20 µm scale (on the left) and a 10 µm scale (on the right) of (**a**) BC6, (**b**) BC4, (**c**) BC11, and (**d**) BC7.

To examine the effect of pyrolysis conditions and KOH activation on the surface morphology of the BCs, they were subjected to SEM analysis. Figure 6 shows the surface morphology of the four BCs. The SEM images display that all samples present sponge-structures with roughness and irregular structure with big holes and cavities, which seem to be more significant for the BC6 and BC4 (see Figure 6a,b), following the SSA and pore structure results. Thus, by the SEM analysis, it is possible to infer that the preparation condition did influence the surface characteristics of the BCs. In addition, the images also show a significant presence of macropores and ultra-macropores, especially in BC11 and BC7. Macropores are also important because if the BCs are used as adsorbents to remove pollutants from waters, they serve as vectors of the solution passage through macropores until it attains smaller pores (in the interior of the BCs). 

#### 2.2.2. Chemical Characterization

**Figure 7:**
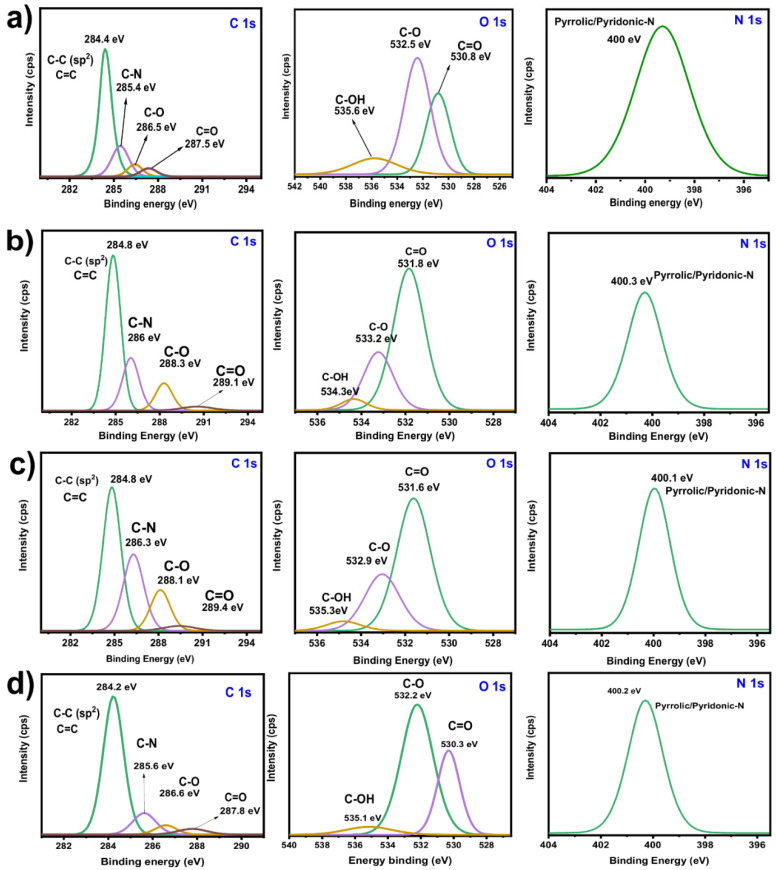
XPS spectra for (**a**) BC6, (**b**) BC4, (**c**) BC11, and (**d**) BC7.

XPS analysis gives valuable information on different surface compositions of BC, which may be caused by the different pyrolysis conditions and oxidative alkaline treatment. Figure 7 shows C 1s, O 1s, and N 1s spectra, corresponding to carbon, oxygen, and nitrogen bonds, respectively. 

The asymmetric C 1s spectra can be deconvoluted into four peaks which are assigned to C-C and C=C (aromatic and aliphatic groups), C-N, C-O, (C-O-C), and (C=O) bonds, as seen in Figure 7. O 1s spectra were deconvoluted in three oxygen–chemical states, which might correspond to oxygen double-bonded with carbon in carbonyl structures (C=O) [27,28], esters (O=C-OC), and carboxyl (O=C-OH) groups [29], as presented in Figure 7. The spectrum for the N 1s exhibits a single peak at around 400 eV that is attributed to pyrrolic/pyridinic nitrogen (graphitic-N species) [30].

The main composition of the BC taken from XPS analysis is shown in Table S2. All selected BCs contain more than 86% of carbon and more than 8.5% of oxygen. The high oxygen content indicates the abundance of functionalities on BCs surfaces. More precisely, the selected BC samples contain between 2 and 3% of carbonyl groups (C=O). The amount of hydroxyl (C-OH) and ether (C-O-C) groups are between 5 and 6%. According to the XPS results, the chemical activation with potassium hydroxide is more effective in increasing the number of functional groups on BCs surfaces. It is well known that the surface functional groups are responsible for improving the adsorption process through electrostatic interactions and chemisorption-based processes [2,3]. 

FTIR was used to examine the presence of the functional groups on the biochar samples (see Figure S1). The results show that the sample BC6, which presented the highest SSA and was prepared with the highest temperature (900 °C), displayed lesser functional groups and peaks with lower intensities (see Figure S1a). This suggests that at high temperatures functional groups can be lost due to the high volatilization of the compounds in the carbonaceous materials. On the other hand, the sample BC7 seems to have more peaks and with higher intensities (see Figure S1d). BC7 was prepared at 700°C.

As can be seen, all samples presented similar spectrums, and the main difference is in the intensity of the bands (see Figure S1); the band at 3462–3473 cm^−1^ are assigned to stretching of O–H groups with intermolecular H bonding [5,9,14], and the small peaks at around 2926–2970 cm^−1^ are related to the asymmetric and symmetric CH- stretching [5,9,14].

The small peaks around 1635–1639 cm^−1^ are related to the stretch of the carbonyl group (C=O) of carboxylic acids. The small bands at around 1429 and 1444 cm^−1^ can be attributed to ring modes of aromatics in the biochar chains [5,9,14]. The bands between 1255 and 1285 cm^− 1^ can be attributed to the C–O stretch of phenols or ethers [5,9,14]. The band at around 1028 and 1034 cm^−1^ are attributed to CO– of alcohols. The small vibrational bands between 791 and 887 cm^−1^ might be assigned to out of plane C–H bends [5,9,14].

**Figure 8:**
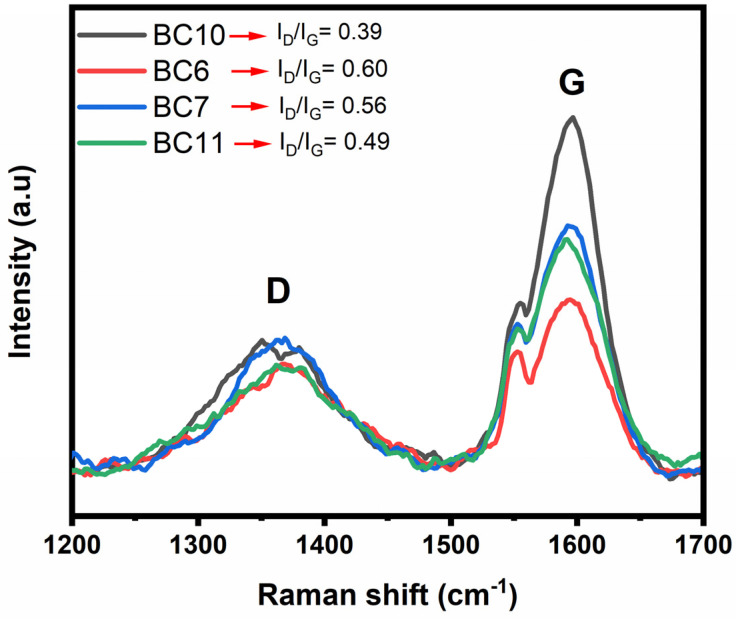
Raman spectra of the BCs.

Raman spectroscopy was performed to evaluate further the structure and degree of graphitization of the BCs. The obtained spectrum and intensity ratio of the four BCs are shown in Figure 8. Two distinct Raman shifts are observed in the spectra. The first one (D-peak), between 1300 and 1450 cm^−1^, is a characteristic scattering peak of graphitic structures. The second one (G-peak), between 1550 and 1650 cm^−1^, is a consequence of lattice defects, disordered arrangement, and the low symmetry carbon structure of graphite [31], characteristic of aromatic rings and hetero rings, especially aromatic amines [32,33]. These two functional groups are the main components of the BCs. The presence of nitrogen in samples is highlighted by XPS analysis.

The degree of graphitization can be evaluated using the ratio of intensities of the D-peaks and G-peaks (I_D_/I_G_) [34]. A smaller I_D_/I_G_ value suggests a higher degree of graphitization. In this sense, the four I_D_/I_G_ ratios are shown in the inset, confirming the resulting material’s graphite structure. The lowest I_D_/I_G_ value reveals the more ordered graphitic structures [34], revealing that BC6 is the more ordered one. It is also the one with the highest SSA. However, it does not seem that the SSA is precisely correlated with the degree of ordination. 

**Figure 9:**
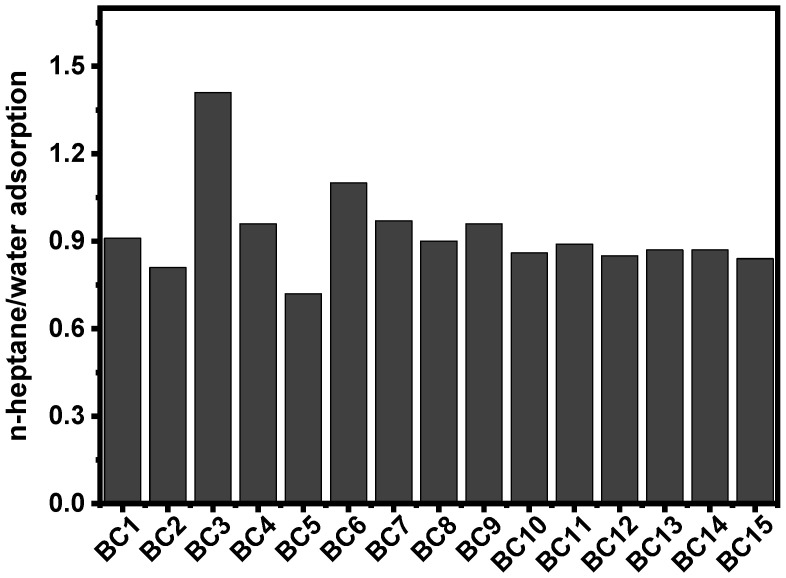
Hydrophobicity/hydrophilicity index of the biochars based on ratio of n-heptane/water adsorption.

Hydrophobic behavior has been studied, and the ratios between n-heptane and water adsorbed by the 15 samples are shown in Figure 9. All samples, except 2, have a ratio below 1, which means that they are more hydrophilic than hydrophobic. These results may prove that the chemical activation by KOH has a significant effect on the surface functional groups of the BCs. Indeed, as carbonaceous materials, it is expected that material is more hydrophobic than hydrophilic. However, the XPS indicated the presence of hydrogen and oxygen groups, which are known to increase the hydrophilicity of the BC samples [4,35]. Thus, hydrophilicity and hydrophobicity could perfectly influence the performance of the BC during the adsorption process through hydrophilic and hydrophobic interactions [9,35]. 

### 2.3. Evans Blue Removal

The adsorption experiments were performed with the BC6 made at 900 °C, 2 h, and biomass: KOH ratio of 1:1—the sample with the highest SSA value (2209 m^2^·g^−1^). Since the adsorption efficiency is highly dependent on the porous carbon textural properties, the highest SSA value justifies the choice of BC10 to be selected to be applied as an active matrix for adsorption.

#### 2.3.1. Effect of pH and Point of Zero Charge

**Figure 10:**
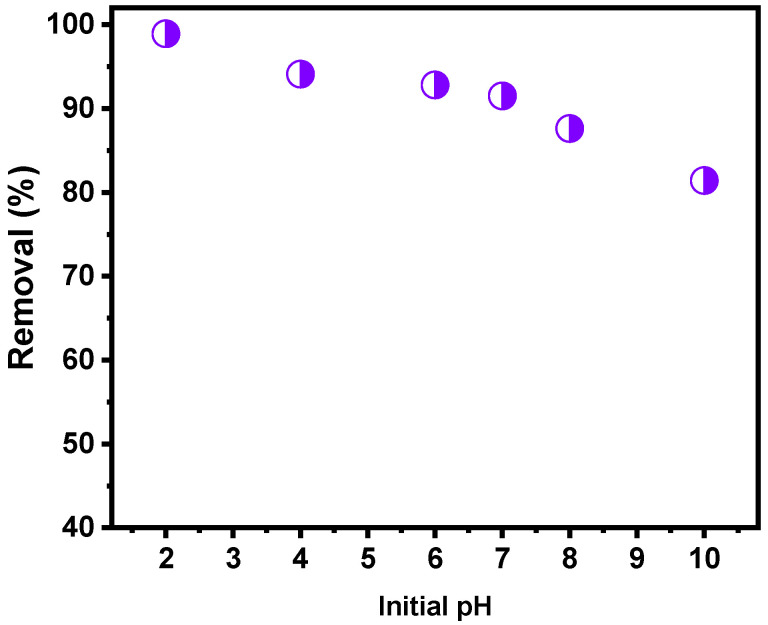
pH effect on the EB dye removal onto BC6. Adsorption experimental conditions: the initial adsorbate concentration was 150 m L^−1^; the contact time was 4 h; the temperature was 23 °C; adsorbent dosage of 1.5 g L^−1^.

The pH of a solution is one of the most crucial parameters that affect the adsorption process [36,37,38,39,40,41,42,43]. The pH effect in the EB removal was performed in the range of 2–10 at initial concentration of 150 mg L^−1^. The results are shown in Figure 10. It seems that the pH played an important role during adsorption of anionic EB dye. Lower percentage removal was shown at basic pH, while at acid pH, the EB removal increased. The lower removal efficiency at higher pH might be because of competitive adsorption between OH− and the anionic EB dye with the biochar surface [36,37,41]. This suggests that the EB removal also takes place due to the electrostatic interactions, since it is dependent of the pH values [36,37,41,42].

Similar results were found by Prola et al. [36] in their study, which employed multiwalled carbon nanotubes and activated carbon to adsorb EB dye. They found that the removal of EB dye decreased with the increase in the pH initial solution. Based on the pH studies, the next adsorption experiments were carried out with pH EB dye solutions at around 7.0 (pH of the prepared EB working solutions), being unnecessary to make any pH adjustments.

To further evaluate the effect of the pH on the EB removal, the point of zero charge of BC6 was determined. The p_HPZC_ value obtained for the BC6 was 6.83 (see Figure S2). For pH values lower than pH_pzc_, the biochar presents a positive surface charge [12,14,36]. The dissolved EB dye is negatively charged in an aqueous solution because it possesses four sulphonate groups [12,14,36]. Then, the EB adsorption is maximized when the biochar has a positive surface charge. Electrostatic interactions occur between biochar and EB at a pH higher than 6.83. However, when the pH value is much lower than pH_pzc_, the surface of the biochar becomes more positive. This phenomenon explains the high adsorption capacity at pH 2.0.

#### 2.3.2. Kinetic Study

The elucidation of the dominant mechanism involved in the adsorption process, such as diffusion control and mass transport processes, is conveniently addressed by kinetics assays. The kinetics of adsorption of EB on selected BC is explored using nonlinear pseudo first-order (PFO), pseudo second-order (PSO), and general-order (GO) kinetic models.

**Figure 11:**
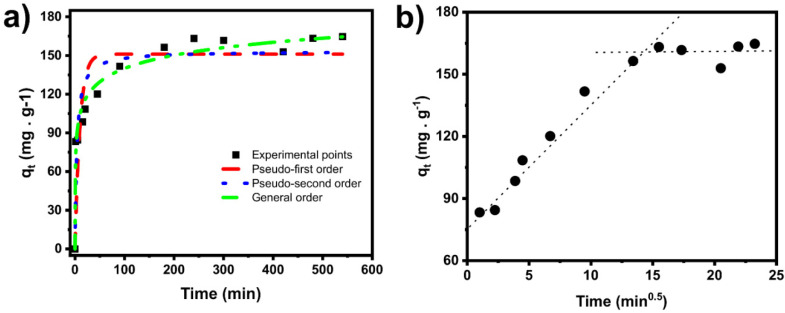
Kinetic fitting models and BC6 experimental curve (**a**) and intraparticle diffusion modeling (**b**).

The kinetic curves and fitting parameters of the models are shown in Figure 11a and Table 4. The suitability of the models was evaluated through the determination coefficient R^2^_adj_ and standard deviation (SD) [42,43,44,45,46,47,48,49]. The general-order model is the most suitable model because it presents the highest R^²^_adj_ and lowest SD values (Table 4). The general-order model suggests that the adsorption order should follow the same trend as that of a chemical reaction [36,44]. Considering the general-order kinetic equation, the order of reaction (*n*) is found to be 33.87, suggesting that EB adsorption processes onto BC6 are complex and need to be further studied.

Since BC6 is highly porous, physical adsorption may play an essential role in the EB adsorption process. To demonstrate, intraparticle diffusion is exhibited in Figure 11b. The adsorption dynamics include two stages. The first stage, the longest one, can be related to boundary diffusion, where the EB is diffused on the BC exterior surface and to the bigger pores [23,36]. In the second stage, the EB is adsorbed and diffused from the bigger to the interior of smaller pores, followed by the equilibrium [23,36].

Further evaluating the kinetic process, t_0.5_ and t_0.95_ were studied. The values were calculated from the best model (general-order model). These values correspond to the times (min) when 50% and 95% of saturation (q_e_) are attained, respectively [22,23,24]. 

Due to BC6’s textural properties and chemical surface features, fast adsorption kinetics are observed from the values of t_0.5_ and t_0.95_. BC6 presented a high SSA and high amount of micropores and mesopores (see Table 2), which can explain the fast and good EB adsorption performance. 

The adsorption procedure was further continued by establishing the contact times of 150 min (2.5 h). The established contact time was slightly higher than the t_0.95_ to ensure that the adsorption process had enough time to reach the equilibrium between EB and BC6, because t_0.95_ will attain 95% saturation; the equilibrium should be established in the condition of complete saturation of the adsorbent.

#### 2.3.3. Isotherm Study

The equilibrium of the adsorption process is one of the most critical pieces of information for the correct understanding of an adsorption process. In addition, it gives reliable information about the adsorption mechanism pathways and effective design of the adsorption system [37]. 

**Figure 12:**
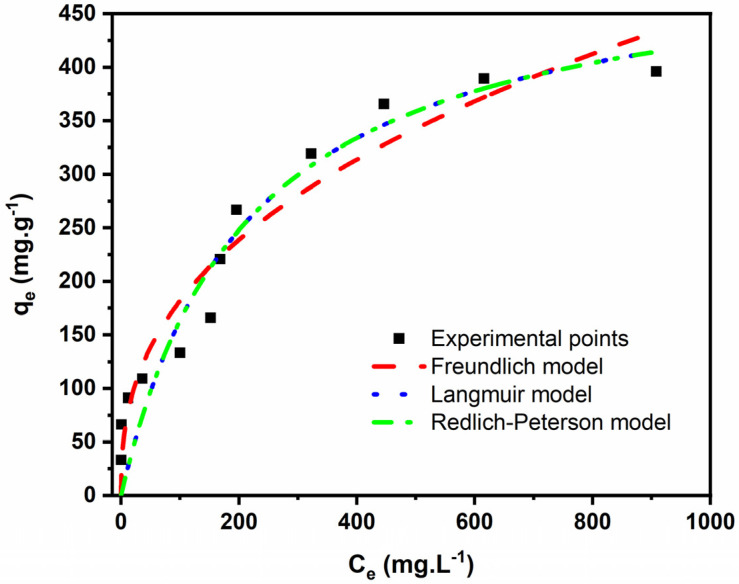
Adsorption isotherms of BC6 and the fitted models.

The equilibrium system between EB and the BC was evaluated using the nonlinear fitting of the Langmuir, Freundlich, and Redlich–Peterson models, and the obtained data are displayed in Figure 12 and Table 5.

Based on that R^2^_Adj_ and SD values, the Freundlich isotherm model was the most suitable model for all three BCs because it presented the highest R^2^_Adj_ and lowest SD values. Freundlich’s model indicates that the adsorption process occurs on heterogeneous surfaces and active sites with different energies (which is the case of our BC) based on multilayer adsorption. For this model, an n value between 0 and 10 suggests favorable adsorption. The n value found in this work was 2.54.

The Freundlich model does not give maximum adsorption value (Q_max_), but the experimental adsorption capacity for the EB onto BC6 was very high (396.1 mg·g^−1^). The physicochemical properties of BC6 can explain the efficiency of EB removal. Besides its very high SSA (2209 m^2^·g^−1^), it also presented a very high pore volume equal to 1.49 cm^3^·g^−1^. It is well known that pore volume plays a decisive role in the overall adsorption, as SSA does [9]. Higher pore volume leads to higher sorption capacity [9]. The EB molecule has a maximum diagonal length of 2.04 nm [36], a size that can be accommodated inside some pores of BC6. 

#### 2.3.4. EB Dye Mechanism of Adsorption on Biochar

**Figure 13:**
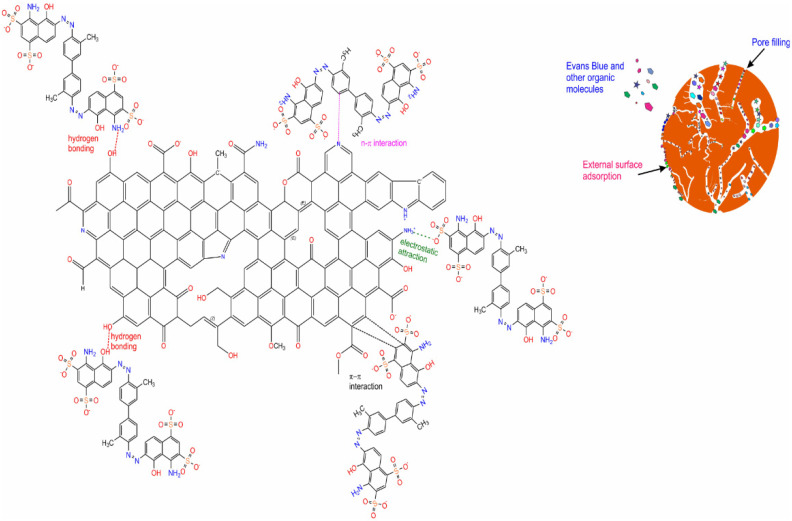
Proposed mechanism of adsorption.

Based on the biochar’s physicochemical characterization, such as SSA, pore size, FTIR, and XPS—as well as the adsorption results, such as the initial pH solution, kinetics of adsorption, and equilibrium studies—it was possible to suggest the primary mechanisms of adsorption for EB on biochar (see Figure 13).

The adsorption process takes place through different physical interactions between biochar surface and EB dye such as hydrogen bonding and π-π and n-π interactions of the aromatic ring of the biochar with the aromatic rings of the EB dye [36,43,48]. Hydrogen bonding is formed with the azo groups of the dye with the hydroxyl groups present on the biochar’s surface [36,43,48]. π-π and n-π interactions (donor–acceptor interactions) occur among aromatic rings in the biochar structure that act as an electron acceptor (see Figure S2). Besides, the aromatic rings of the EB dye molecules interact with the C=O, OH, COOH, and phenyl groups of the biochar that act as adsorption sites (see Figure 13) [36,43,48]. 

However, since the biochar has highly developed porosity and elevated SSA, another mechanism that takes place is the pore-filling. The pore-filling can be the most prominent process that contributes to the high adsorption efficiency for EB dye onto highly porous biochar (see Figure 13).

#### 2.3.5. EB Adsorption Performance over Norway Spruce BC and Other Adsorbents: Comparison with the Literature

The adsorption tests strongly indicated that Norway spruce bark BC efficiently removed EB from aqueous solutions. Although the nature of every adsorbent is different and each absorbent has its own merits and demerits, here, we have provided comparison data in which the adsorbent dose, pH, and maximum adsorption capacity are taken into consideration. The values were obtained at the best experimental conditions of each work. As a result, it can be seen that the adsorption efficiency of the EB molecules on Norway spruce BC was very high in comparison with other listed adsorbents in Table 6.

It is worth discussing that, among the reported adsorbents, the synthesized BC exhibited the third-highest sorption capacity; however, the first one (multiwalled carbon nanotube) and the second one (rarasaponin–bentonite) are materials that present more complex preparation methodologies; therefore, higher costs are involved in comparison with a single step KOH BC process. Consequently, the BC can be considered a cost-efficient adsorbent due to its facile fabrication process being an effective prototype from the environmental perspective for removing azo dyes and possibly other organic pollutants from wastewater.

#### 2.3.6. Synthetized Wastewater Treatment Tests

**Figure 14:**
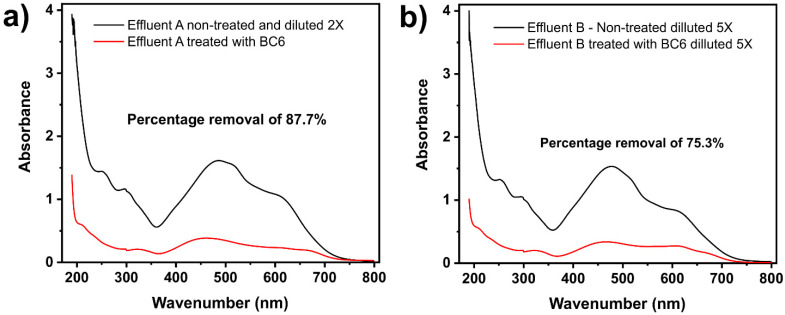
Spectra of effluents A (**a**) and B (**b**) treated and non-treated by BC10.

As previously observed, sample BC6 was very efficient in removing EB from aqueous solutions. Consequently, it is expected that it could be effectively employed in the treatment of wastewaters composed of compounds commonly found in industrial wastewaters. Therefore, two synthetic wastewaters loaded with seven dyes and other organic and inorganic compounds (see Table S1) were employed to test the ability of the BC10 to clean them up (see Figure 14). 

The calculation of the percentage removal was performed taking into account the UV–vis spectra area of the two synthetic effluents before and after the treatment under the band of absorption from 190 to 800 nm [43,44,48] (see Figure 13). 

The spectra show very high removal percentages for both effluents: 87.7% for effluent A (low concentration) and 75.5% for effluent B (highly concentrated). These results strongly support the practical application of the KOH-activated Norway spruce bark in treating real colorful wastewater.

#### 2.3.7. Regeneration Studies

The reuse of the biochar after the adsorption process makes them even more important, given their low cost and their ability to regenerate. It is crucial for environmental and cost reasons for adsorbents to have good recyclability. Usually, this is carried out by leaching-out the adsorbed dye with alcohol or a basic solution with a pH at which dye adsorption is very low.

**Figure 15:**
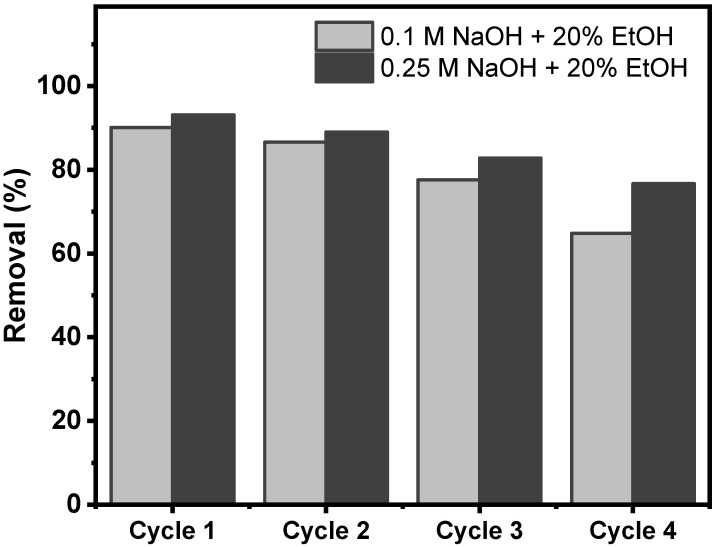
Effect of the eluent on the desorption and adsorption–desorption cycles.

The BC6 sample was subjected to four adsorption–desorption cycles. The tests were performed at EB dye initial concentration of 200 mg L^−1^, an adsorbent dosage of 1.5 g L^−1^. Two eluents were put in contact with the biochar loaded with EB dye (solutions of 0.1M NaOH + 20% EtOH and 0.25M NaOH + 20% EtOH) (see Figure 15).

The results depicted in Figure 15 suggests impressive adsorption performance after five continuous adsorption–desorption cycles of the BC6, for both eluents. It was observed that the adsorption capacity of the BC6 kept at very high level, with a reduction of 4.1%, 10.3%, and 16.4% after second, third, and fourth cycles, respectively, for the eluent 0.25M NaOH + 20% EtOH.

As the main takeaway of the regeneration results, the biochar exhibited very good reusability even after a fourth cycle. Thus, further experiments on testing different eluents could help the biochar to reach even higher adsorption performances after four cycles.

## 3. Materials and Methods

### 3.1. Chemicals and Reagents

The Holmen paper industry provided the Norway spruce bark. The spruce bark was dried and milled at a medium particle size of 500 µm with a Fritsch Pulverisette 14 miller. The chemicals potassium hydroxide (KOH), Evans blue dye (C_34_H_24_N_6_Na_4_O_14_S_4_), and hydroxide chloride (HCl) were purchased from Sigma-Aldrich. The chemical structure of the Evans blue dye is shown in Figure S3.

### 3.2. Preparation Process

The BCs were prepared by following the method described as follows: First, 15.0 g of bark was mixed with KOH at different ratios (biomass: KOH, 1:1, 1:1.5, and 1:2, weight), and mixed with 30 mL of distilled water in a melting pot, until a homogeneous paste is obtained. The mixtures were left for 2 h at ambient temperature and then placed in a stove, at 105 °C, for a day before being pyrolyzed following the RSM procedure. The pyrolysis occurred in a Carbolite Gero Elf 11/23 chamber furnace under a 600 cm^3^ min^−1^ nitrogen flow at different pyrolysis conditions. After pyrolysis, the samples were milled and washed with 1.0 M hydrochloric acid solution to remove the remaining chemical reagent. The washing step was performed using a proportion of 100 mL of HCl to 15 g of pyrolyzed sample. Both BC and HCl solution was placed in a flat-bottomed flask under a reflux system at 75 °C for 1 h and magnetically stirred at 300 rpm [4,5]. Afterward, the acid solution was separated from the solid samples by filtration. In order to complete the BC preparation, a washing step with distilled water was performed several times until the pH value of the filtrate reached a neutral value [4,5]. The wet BCs were finally drought in a 100 °C stove over the night for further uses. 

### 3.3. Response Surface Methodology (RSM)

In order to obtain BCs with optimized properties, the influence of pyrolysis variables (pyrolysis temperature, holding time, and ratio of activating agent) was studied (see Table 1). 

The RSM experiments were designed using a Box–Behnken design (BBD). A 3-variables, level Box–Behnken design, consisting of 15 experimental runs, was adopted to optimize the experimental data, including 3 replications at the center point, as shown in Table 1. The plan was automatically generated using Minitab software, which was explored to verify the variables’ influence on the responses (SSA and mass yield).

#### BC Characterization

The textural properties of BCs were evaluated through nitrogen adsorption–desorption isotherms by using a Tristar 3000 apparatus, Micrometrics Instrument Corp. The BC samples were subjected to degasification at 180 °C for 3 h in a nitrogen atmosphere. The specific surface area (SSA) and pore size distribution were obtained using the Brunauer–Emmett–Teller (BET) method [8].

The morphology of BCs was studied through scanning electron microscopy (SEM) using a Zeiss-Gemini microscope, and pictures are made at 10 µm and 20 µm scales. 

XPS spectra were collected using a Kratos Axis Ultra DLD electron spectrometer using a monochromated Al K_α_ source operated at 150 W. An analyzer of 160 eV for acquiring survey spectra and 20 eV for individual photoelectron lines were used. The samples were gently hand-pressed using a clean Ni spatula into the powder sample holder. Because carbon material is conductive, no charge neutralization system was used. The binding energy (BE) scale was calibrated following the ASTM E2108 and ISO 15472 standards. Processing of the spectra was accomplished with the Kratos software.

Fourier transform infrared spectroscopy (FTIR) was exploited to determine the functional groups of the biochars. The FTIR spectra were recorded over the wavenumber range of 4000–400 cm^−1^, utilizing a Bruker IFS 66v/S instrument (Bruker Optics, Ettlingen, Germany) with an acquisition of 64 scans min^−1^ and resolution of 4 cm^−1^.

Raman spectra were collected using a Bruker Bravo spectrometer (Bruker, Ettlingen, Germany) connected to a docking measuring station. Shortly, 0.5 g of BC samples was manually ground using an agate mortar and pestle, placed in 2.5 mL glass vials, and scanned in the 300–3200 cm^−1^ spectral range at 4 cm^−1^ resolution for 256 scans. Min–max normalization over the 1000–2000 cm^−1^ region and smoothing (9 points) was carried out using the built-in functions of the OPUS software (v7, Bruker Optik GmbH, Ettlingen, Germany). No baseline correction was needed.

The hydrophobicity index (HI) was obtained according to a method reported by [42]: 0.3 g of each BC was added in 10 mL beakers and placed into plugged 1.5 L E-flasks with saturated atmosphere solvent vapor, 80 mL of each solvent (n-heptane or water). After 24 h, the beakers were weighed. The weight gained was employed to calculate the adsorbed vapor sorption.

The pH_pzc_ values were obtained by the relation between the initial pH_i_ and the variation of pH (pH_f_–pH_i_) [33,36].

### 3.4. Evans Blue (EB) Removal Process

#### 3.4.1. Batch Adsorption Tests

The adsorption tests were performed according to the reported in [9,42,43,44]. The EB stock solution of 2000 mg·L^−1^ was diluted in several solutions in 50.0 to 1500 mg·L^−1^. First, 30 mg of BC were weighted in 50 mL Falcon tubes and put in contact with 20 mL of EB solution (1.5 g·L^−1^). Then, the tubes were agitated in a shaker model TE-240 from 1 to 540 min. Afterward, to separate the BCs from the EB solutions, the tubes were subjected to centrifugation at 5000 rpm for 10 min. Next, the supernatants were collected and placed into smaller tubes of 15 mL and centrifuged again at 5000 rpm for 20 min. Afterward, the residual EB was identified from curves in the UV spectrophotometer Shimadzu 1800 at 607 nm. 

The quantification of the EB adsorbed was calculated through Equation (1) (percentage of removal) and Equation (2) (adsorption capacity), as follows:

(1) $$\%\;\mathrm{Removal}=100\cdot \frac{\left(C_{i}-C_{f}\right)}{C_{0}}$$

(2) $$q=\frac{\left(C_{i}-C_{f}\right)}{m_{\mathrm{BC}}}\cdot V_{\mathrm{pollutant}}$$

q is the removal capacity of adsorbed EB by the BCs (mg·g^−1^). C_i_ is the EB initial solution concentration in contact with the BCs (mg·L^−1^). C_f_ is the EB final concentration after adsorption (in mg·L^−1^). m_BC_ is the mass of BCs (g). V_pollutant_ is the aliquot of the EB solution (L) introduced in the flask.

#### 3.4.2. Kinetic and Isotherm Models of Adsorption

Pseudo first-order (PFO)—see Equation (3)—pseudo second-order (PSO)—see Equation (4)—and general-order (GO) models—see Equation (5)—were used to fit the kinetic data [43,44,45]. The corresponding equations of these respective models are summarized as follows:

(3) $$q_{t}=q_{e}\cdot \left[1-\exp\left(-k_{1}\cdot t\right)\right]$$

(4) $$q_{t}=\frac{k_{2}\cdot q_{e}^{2}\cdot t}{1+q_{e}\cdot k_{2}\cdot t}$$

(5) $$q_{t}=q_{e}-\frac{q_{e}}{{\left[k_{N}\cdot {\left(q_{e}\right)}^{n-1}\cdot t\cdot \left(n-1\right)+1\right]}^{1/\left(n-1\right)}}$$

where t is the contact time (min); q_t_, q_e_ are the amount of adsorbate adsorbed at time t and the equilibrium (mg·g^−1^), respectively; k_1_ is the pseudo first-order rate constant (min^−1^); k_2_ is the pseudo second-order rate constant (g.mg^−1^.min^−1^); k_N_ is the general-order rate constant ((mg·g^−1^)^n−1^·min^−1^); n is the order of the general-order model (dimensionless).

Langmuir’s (Equation (6)), Freundlich’s (Equation (7)), and Redlich–Peterson’s (Equation (8)) models were employed for evaluating the EB equilibrium process [44], as follows:

(6) $$q_{e}=\frac{Q_{\max}\cdot K_{L}\cdot C_{e}}{1+K_{L}\cdot C_{e}}$$

(7) $$q_{e}=K_{F}\cdot C_{e}^{1/\mathrm{nF}}$$

(8) $$q_{e}=\frac{K_{\mathrm{RP}}\cdot C_{e}}{1+a_{\mathrm{RP}}\cdot C_{e}^{\beta }}\;,\;\mathrm{being}\;0\leq \beta \leq 1$$

where q_e_ is the adsorbate amount adsorbed at equilibrium (mg·g^−1^); C_e_ is the adsorbate concentration at equilibrium (mg·L^−1^); Q_max_ is the maximum sorption capacity of the adsorbent (mg·g^−1^); K_L_ is the Langmuir equilibrium constant (L·mg^−1^); K_F_ is the Freundlich equilibrium constant (mg·g^−1^·(mg·L^−1^)^−1/n^_F_); K_RP_ and a_RP_ are the Redlich–Peterson equilibrium constant ((mg·g^−1^)·(mg·L^−1^)^−1^ and (mg·L^−1^)^−^^β^); n_F_ and β are the exponents of Freundlich and Redlich–Peterson models, respectively (dimensionless).

The fitting of the kinetics and equilibrium data were evaluated by using nonlinear methods, which were provided by the Simplex method and the Levenberg–Marquardt algorithm using the fitting facilities of the Microcal Origin 2020 software [45,46,47]. The suitableness of the kinetic and equilibrium models was evaluated using the determination coefficient (R²), the adjusted determination coefficient (R²_ad_j), and the standard deviation of residues (SD) [45,46,47], as shown in Equations (9)–(11), respectively:

(9) $$R^{2}=\left(\frac{\sum_{i}^{n}{\left(q_{i,\exp}-{\bar{q}}_{i,\exp}\right)}^{2}-\sum_{i}^{n}{\left(q_{i,\exp}-q_{i,\mathrm{model}}\right)}^{2}}{\sum_{i}^{n}{\left(q_{i,\exp}-{\bar{q}}_{i,\exp}\right)}^{2}}\right)$$

(10) $$R_{\mathrm{adj}}^{2}=1-\left(1-R^{2}\right).\left(\frac{n-1}{n-p-1}\right)$$

(11) $$\mathrm{SD}=\sqrt{\left(\frac{1}{n-p}\right).\overset{n}{\underset{i}{\sum }}{\left(q_{i,\exp}-q_{i,\mathrm{model}}\right)}^{2}}$$

In the above equations, q_i model_ is the theoretical q value predicted by the model; q_i, exp_ is the experimental q value; ${\bar{q}}_{i,\exp}$ is the average of all measured experimental q values; n is the number of experiments; p is the number of parameters in the fitting model. Values of R^²^_adj_ and SD are used to compare different models of kinetics and equilibrium presented in this work. The best fitting model would present the highest R^²^_adj_ and lowest SD values [45,48,49,50]. Lower SD and higher R^2^_adj_ values show a reduced disparity between experimental and theoretical q values and, therefore, a higher suitability of the model [45,48,49].

#### 3.4.3. Application to Synthetized Effluents 

Two effluents were made, mixing seven dyes and different organic and inorganic compounds to simulate dye-composed effluents. The different compositions of these synthesized effluents are reported in Table S1. In order to prove the ability of the activated carbon for treating real effluents, a calculation of the percentage removal is performed, considering the UV–vis spectra area of the two effluents before and after the treatment under the band of the spectra [43].

## 4. Conclusions

Norway spruce bark BC, through KOH activation, yielded highly porous BC structures. The application of a BBD successfully optimized the production of BC with SSA up to 2209 m².g^−1^. Pyrolysis temperature was the only influential parameter over both responses (SSA and mass yield) within the parameters studied. The temperature influenced the SSA values positively, while the mass yield influenced negatively. In other words, the higher the SSA, the lower the yield. 

The preparation process conditions influence the physicochemical properties of BC. The characterization results indicated that the BCs exhibited disordered carbon structures and presented a high quantity of O-containing functional groups on their surfaces, which improved adsorption performance towards dyes removals. 

The adsorption of Evans blue dye was tested with the BC with the highest SSA, and the kinetic study suggested that the general-order model best fitted the process. Furthermore, the intraparticle process indicated two stages in the EB adsorption process.

The equilibrium study suggested that the EB removal was better described by the Redlich–Peterson model, indicating that the process combines a monolayer and an infinite multilayer adsorption process. The highest adsorption capacity reached is 396.1 mg·g^−1^. The employment of the BC in the treatment of synthetic effluents, with several dyes and other organic and inorganic compounds, returned a high percentage of removal degree up to 87.7%.

Desorption and cyclability tests showed that the biochar can be efficiently regenerated maintaining an adsorption capacity of 75% after four adsorption–desorption cycles.

The very high SSA, high surface functionalities, chemical structure, and highly efficient EB adsorption capacity and effluents removal of the BCs show great potential for using Norway spruce bark as a precursor for the BC preparation with good results adsorption properties.

## Figures and Tables

**Table 1:** BBD matrix of Box–Behnken surface response methodology.

Experiment Samples	Coded Samples	Temperature (°C)	Holding Time (h)	Ratio	SSA (m²·g^−1^)	Mass Yield (%)
700:1:1.5	BC1	700	1	1.5	346	23.6
900:1:1.5	BC2	900	1	1.5	1355	3.0
700:3:1.5	BC3	700	3	1.5	557	21.3
900:3:1.5	BC4	900	3	1.5	1812	3.9
700:2:1	BC5	700	2	1	726	19.3
900:2:1	BC6	900	2	1	2209	11.3
700:2:2	BC7	700	2	2	274	36.8
900:2:2	BC8	900	2	2	418	10.2
800:1:1	BC9	800	1	1	754	12.1
800:3:1	BC10	800	3	1	1415	8.2
800:1:2	BC11	800	1	2	572	11.5
800:3:2	BC12	800	3	2	1434	5.0
800:2:1.5	BC13	800	2	1.5	2011	10.6
800:2:1.5	BC14	800	2	1.5	2002	12.7
800:2:1.5	BC15	800	2	1.5	1943	10.7

**Table 2:** Short literature review of ACPB preparation and SSA values.

Biomass	Temperature (°C)	Holding Time (h)	Ratio	SSA (m²·g^−1^)	Ref.
Norway spruce bark	900	2	1:1	2209	This work
Oleaster fruits flesh	800	1	1:3	1816	[21]
Spent tea leaves	800	1	1:1	820.7	[22]
Oak wood sawdust	800	1	1:0.5	1662	[23]
Amazonian nut shells	800	1	1:1	1624	[24]

**Table 3:** Microporosity and mesoporosity data of the four selected samples.

Sample’s Name	S_meso_ (m^2^·g^−1^)	S_micro_ (m².g^−1^)	S_meso%_ (%)	S_micro%_ (%)
BC6	499	1710	22.6	77.4
BC4	825	1061	43.7	56.3
BC11	99	627	13.6	86.4
BC7	124	150	45.3	54.7

**Table 4:** Kinetic fitting parameters.

Kinetic Models	
Pseudo first-order model	
q_1_ (mg g^−1^)	148.7
k_1_ (min^−1^)	0.09438
R^2^	0.5832
R^2^_adj_	0.5534
SD (mg g^−1^)	31.73
Pseudo second-order model	
q_2_ (mg g^−1^)	158.7
k_2_ (g mg^−1^ min^−1^)	9.602 × 10^−4^
R^2^	0.7209
R^2^_adj_	0.7010
SD (mg g^−1^)	25.96
General-order model	
q_n_ (mg·g^−1^)	630.0
kn ((g·mg^−1^)^n−1^·min^−1^)	4.420 × 10^−28^
n (-)	33.87
R^2^	0.9681
R^2^_adj_	0.9617
t_0.5_	1.5
t_0.95_	139.4
SD (mg g^−1^)	432.0

**Table 5:** Isotherm fitting parameters.

Isotherm Models	
Langmuir	
Q_max_ (mg g^−1^)	511.5
K_L_ (L mg^−1^)	0.004700
R^2^	0.9198
R^2^_adj_	0.9117
SD (mg g^−1^)^2^	39.08
Freundlich	
K_F_ ((mg g^−1^)(mg L^−1^)^−1/nF^)	29.66
n_F_	2.540
R^2^	0.9318
R^2^_adj_	0.9250
SD (mg g^−1^)^2^	36.02
Redlich–Peterson	
K_RP_((mg·g^−1^)·(mg·L^−1^)^−1^)	2.403
a ((mg·L^−1^)^−b^)	0.004700
B	1.000
R^2^	0.9198
R^2^_adj_	0.9019
SD (mg g^−1^)	41.19

**Table 6:** Comparison of EB adsorption capacity on Norway spruce bark BC and other parameters obtained from the different materials reported in the literature.

Biomass	Dosage (g·L^−1^)	pH	Isotherm Model	Kinetic Model	Q_max_ (mg·g^−1^)	Ref.
Magnetic spinel ZnFe_2_O_4_ nanomaterial	0.2	7.0	Freundlich	Pseudo Second Order	45.45	[19]
Commercial activated carbon	1.5	2.0	Liu	General-order	135.2	[36]
Multiwalled carbon nanotube	1.5	2.0	Liu	General-order	409.4	[36]
Rarasaponin–bentonite	10.0	-	Toth	Pseudo First Order	495.8	[38]
Calcined Cu-Al-CO_3_ layered double hydroxide materials	4.0	6.0	Langmuir	-	333.3	[39]
Perovskite lanthanum aluminate nanoparticles	0.6	7.0	Langmuir	pseudo-second-order	40.82	[40]
Mg-Al-CO_3_	0.5	6.0	langmuir		107.5	[41]
Aqai palm stalk (Euterpe oleracea)	2.5	2.0	Sips	Avrami fractional order	45.1	[43]
Norway spruce bark BC	1.5	7.0	Redlich–Peterson	General-order	396.1 *	This work

* Experimental q, Redlich–Peterson model does not provide Q_max_.

## Data Availability

Not applicable.

## Supplementary Materials

The following supporting information can be downloaded. Table S1: Effluents’ composition, Table S2: XPS elemental composition of the biochar samples (atom %), Figure S1: FTIR absorption spectra of (a) BC6, (b) BC4, (c) BC11 and (d) BC7 samples, Figure S2: Point of zero charge curve of the BC6 sample, Figure S3: A- Structural formula of Evans blue dye; B- Optimized three-dimensional structural formula of DB-53. The dimensions of the chemical molecule was calculated using ChemBio 3D Ultra version 12.0.

## Sample Availability

Samples of the compounds are available from the authors.
