# Supplementary material for: Process Parameters Optimization, Characterization, and Application of KOH-Activated Norway Spruce Bark Graphitic Biochars for Efficient Azo Dye Adsorption
Source: Molecules. 2022 Jan 11;27(2):456. doi: 10.3390/molecules27020456 (PMC8780614; doi:10.3390/molecules27020456)
Supplement: Supplementary file 1 [file molecules-27-00456-s001.zip › molecules-1530524-supplementary.pdf]

Supplementary data

Table S1. Effluents' composition

Compounds	Concentration (mg L^{-1})		λ_{max} (nm)
Effluent	A	B	
Evans blue	40	80	607
Reactive orange 16	20	40	494
Reactive blue 4	20	40	595
Methyl Red	20	40	507
Bismarck Brown	20	40	468
Methylene Blue	20	40	668
Methyl Orange	20	40	522
Sodium Dodecyl	20	40	-
Sodium sulfate	20	40	-
Urea	10	20	-
Ammonium chloride	15	30	-
Sodium acetate	15	30	-

Table S2. XPS elemental composition of the biochar samples (atom %)

			O1s	
Samples	C1s	O1s	C=O	C-OH, C-O-C
BC6	86.6	10.5	2.8	5.6
BC4	87.2	9.1	2.6	5.0
BC11	89.9	8.9	2.1	5.4
BC7	89.4	9.3	2.4	5.2

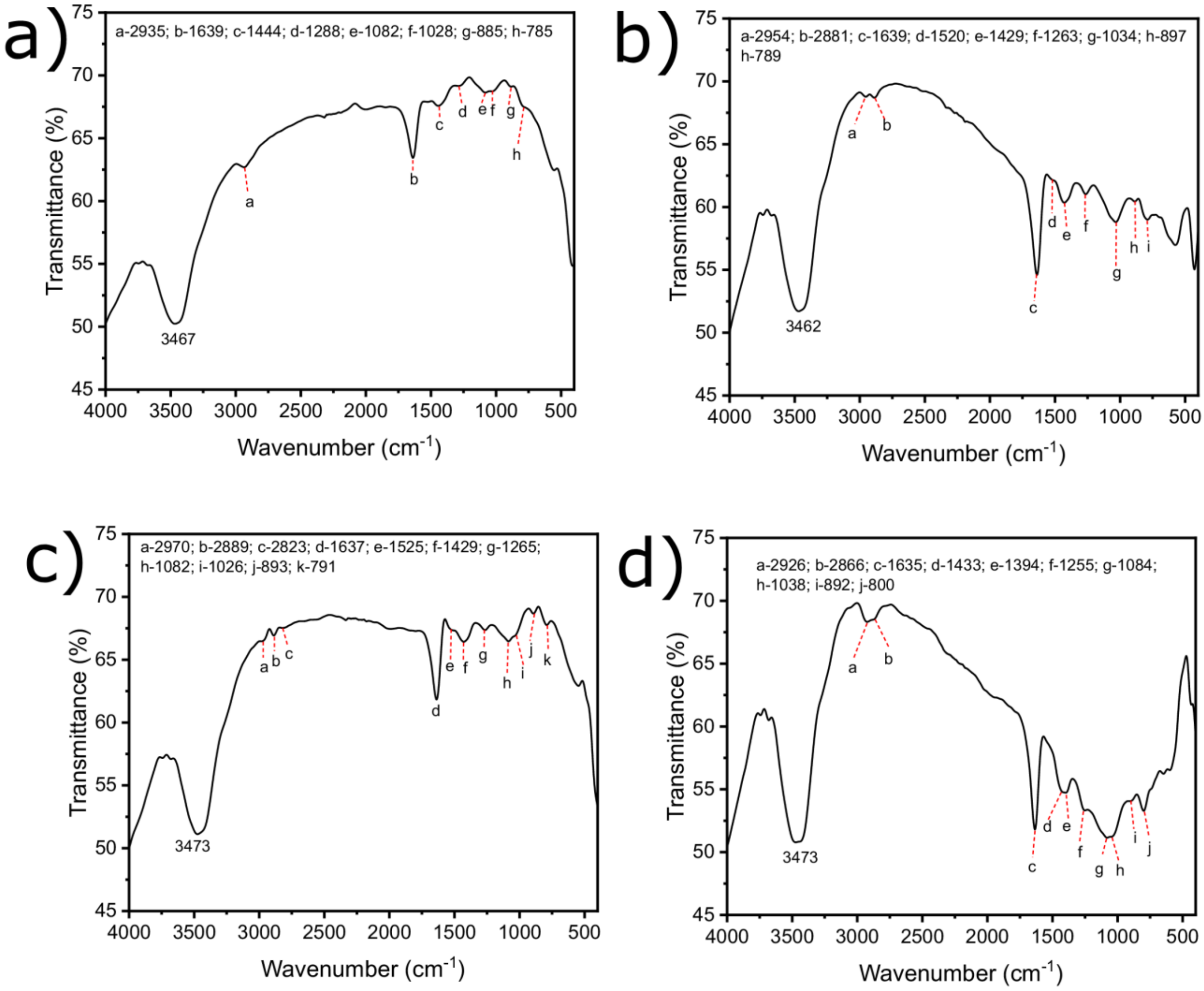

Figure s1. FTIR absorption spectra of (a) BC6, (b) BC4, (c) BC11 and (d) BC7 samples.

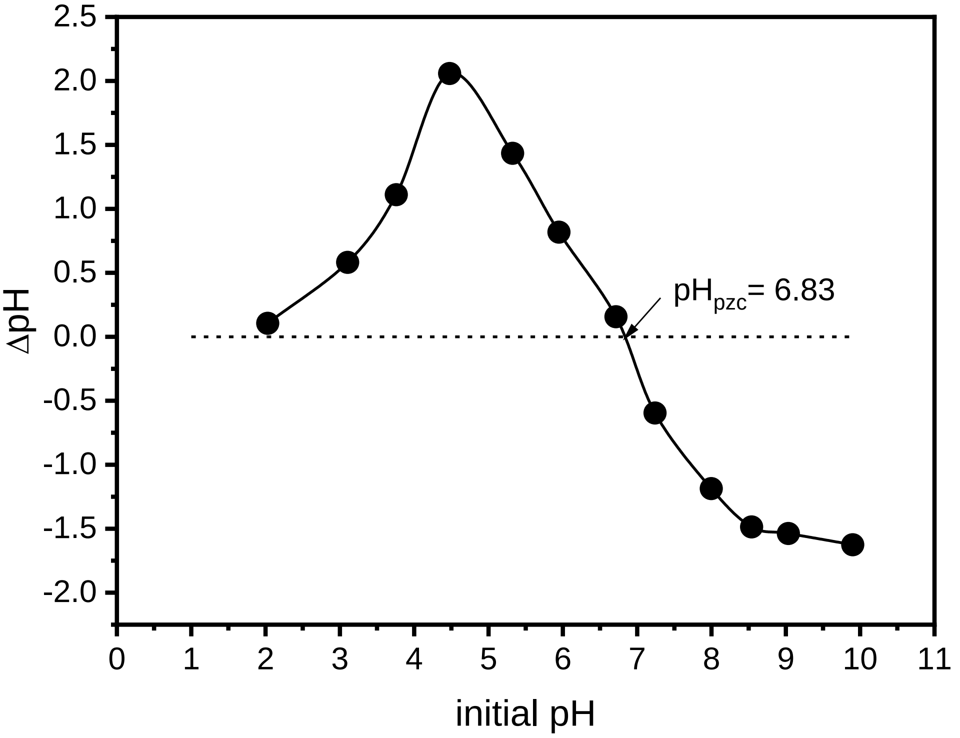

Figure S2. Point of zero charge curve of the BC6 sample.

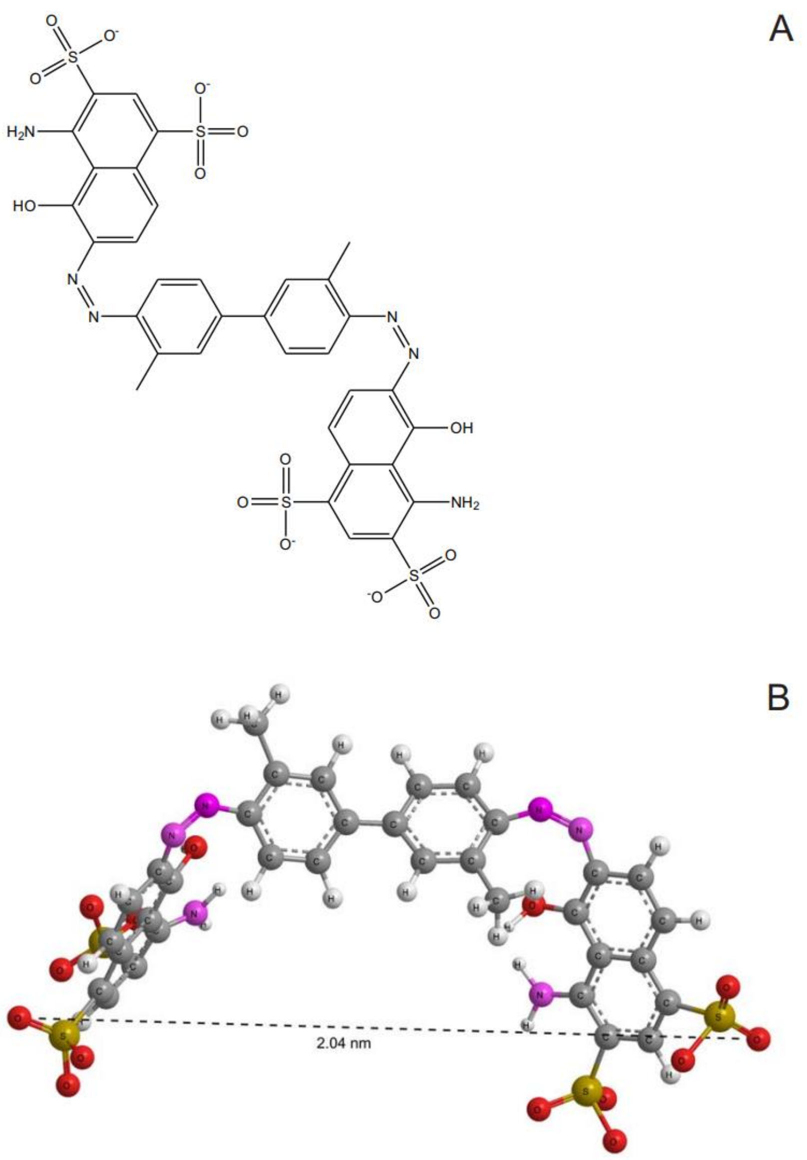

Figure S3. A- Structural formula of Evans blue dye; B- Optimized three-dimensional structural formula of DB-53.The dimensions of the chemical molecule was calculated using ChemBio 3D Ultra version 12.0.
